# AFM for Studying the Functional Activity of Enzymes

**DOI:** 10.3390/biom15040574

**Published:** 2025-04-12

**Authors:** Irina A. Ivanova, Anastasia A. Valueva, Maria O. Ershova, Tatiana O. Pleshakova

**Affiliations:** Institute of Biomedical Chemistry, Pogodinskaya Str., 10, 119121 Moscow, Russia; i.a.ivanova@bk.ru (I.A.I.); varuevavarueva@gmail.com (A.A.V.); motya00121997@mail.ru (M.O.E.)

**Keywords:** atomic force microscopy, single-molecule enzymology, protein structure, protein function, AFM imaging

## Abstract

The conventional approach to investigating enzyme systems involves the simultaneous investigation of a large number of molecules and observing ensemble-averaged properties. However, modern science allows us to study the properties of single molecules and to obtain data on biochemical systems at a fundamentally new level, significantly expanding our understanding of the mechanisms of biochemical processes. Imaging of single biomolecules with high spatial and temporal resolution is among such modern research tools. To effectively image the individual steps or intermediates of biochemical reactions in single-molecule experiments, we need to develop a methodology for data acquisition and analysis. Its development will make it possible to solve the problem of separating the static and dynamic disorder present in the parameters identified by traditional proteomic methods. Such a methodology may be based on AFM imaging, the high-resolution microscopic visualization of enzymes. This review focuses on this direction of research, including the relevant methodological and practical solutions related to the potential of developing a single-molecule approach to the study of enzyme systems using AFM-based techniques. We focus on the results of enzyme reaction studies, as there are still few such studies, as opposed to the AFM studies of the mechanical properties of individual enzyme molecules.

## 1. Introduction

The study of kinetic processes at the single-molecule level is a relatively recent direction in modern biochemistry. According to statistics from the PubMed platform, more than 400 papers on this topic (retrieved using the keyword string “single-molecule enzymology”) have been published worldwide in the last five years, which is an indication of its relevance. This is a promising trend due to the potential to use the resulting knowledge to diagnose diseases associated with enzyme dysfunction [1,2]. Research into kinetic activity lies at the interface of different domains of science, from classical enzymology to single-molecule biophysics. The key advantage of using biophysical methods to study kinetic processes at the single-molecule level is that they allow one to investigate the heterogeneity of free energy states in molecular populations, which is generally a challenging problem for conventional ensemble averaging approaches [3]. Brownian fluctuations and thermal forces are factors playing an important role in molecular heterogeneity. They are the main source of noise and variability in single-molecule experiments. In many cases, it is difficult to differentiate between molecular heterogeneity and stochastic noise caused by thermal effects. A system can be characterized by both temporal and spatial heterogeneity; single-molecule studies allow these differences to be tracked. In cases when the ensemble methods allow one to obtain mean values only, the techniques enabling single-molecule detection may eventually provide not only the mean value but also the probability distribution on both sides of this mean value. The distribution of values with distinct clusters separated by gaps may indicate different energy or conformational states, while the position of the mean value relative to two distinct clusters may indicate that a certain state is preferred over another. The probability distribution of values may detect deviations from the statistical mean behavior and, with sufficient biological and physical insight, become a basis for investigating the potential mechanism of the observed behavior that lies outside the scope of what can be deduced from the simple average value obtained using the integral method [4].

Single-molecule studies of enzyme systems allow the observation of transient states and intermediate products for which information can be lost during ensemble measurements. It is possible to determine the dependence between the mechanistic movements of a globule and the catalytic function within a single molecule. It is clear that in enzymatic reactions for which the activity is measured using the conventional ensemble method (e.g., as the number of enzymatic cycles per unit of time), the functions of the molecules that make up the system as a whole are not synchronized. In other words, at each specific moment, each enzyme molecule can undergo a different stage of the reaction. In the meantime, chemical reactions carried out in a single-molecule system give an idea of the fluctuations in properties that are disguised when the properties of a molecular ensemble are measured [5]. Thus, there are models that allow one to determine the distribution of a property over time in simple extreme cases rather than an averaged value, as well as study their sensitivity to the initial conditions [6]. The biochemical sense of a catalytic reaction at the single-molecule level has been demonstrated by both computational and experimental studies, which have shown that the Michaelis–Menten equation is still valid even for an enzyme but has a different microscopic interpretation [7].

Advances in experimental and computational methods have spurred the emergence of integrated tools that can be used in research related to stubborn biological problems. Experimental progress has been achieved due to the enhancement of the sensitivity and operating speed of sensors, the stability and efficiency of light sources, probes, and microfluidic devices, as well as important factors such as improvements in the mathematical methods underlying the operation of the equipment [4]. Biophysical methods have already been used in single-molecule studies from the perspective of common soft condensed matter [8], as well as complex nanosized biomolecular machines [9] and features of the behavior of individual molecules in living cells [10,11].

The key characteristics of enzyme-catalyzed reactions have been extensively studied at the single-molecule level using a variety of physicochemical methods. Protein structures and functions have been determined, but most of them have been reported for the static molecular conformation. Thus, a lot of information on protein structure has been obtained using techniques such as small-angle X-ray scattering [12] and nuclear magnetic resonance (NMR) [12,13,14]. The dynamic investigation of the properties of analyzed molecules during reactive events, both in simple single-step reactions and in complex multistep processes, is a relevant problem. A large body of data has been obtained using techniques such as total internal reflection fluorescence microscopy (TIRFM), confocal microscopy, single-molecule fluorescence resonance energy transfer (smFRET), fluorescence correlation spectroscopy (FCS), optical tweezers (OTs), and magnetic tweezers (MTs) [15].

Combinations of methods such as correlative atomic force microscopy and fluorescence microscopy are often used for measurements in environments that are closest to physiological ones. Single-molecule fluorescence analysis has provided valuable data on the correlation between changes in the rate of a catalytic reaction and the conformational fluctuations of the enzyme (e.g., [7]). Fluorescence microscopy techniques that are used for the study of enzymatic reactions lie beyond the scope of this review. For fluorescence-based methods, it is required to insert specific labels in which dyes are covalently linked after site-directed mutagenesis, which affects the native structure of protein molecules. Furthermore, the processes that occur during label staining limit the time range of any particular experiment to three orders of magnitude or less, although single-photon detection has a nanosecond temporal resolution, and fluorescence experiments can technically last for hours [16,17].

The interactions between objects are force-driven, so they are key parameters in biological mechanisms ranging from physiological to cellular and molecular processes such as cotranslational folding, sensory reception, adhesion, and cohesion [18,19]. Processes such as protein folding, translocation, substance transport, and biomolecular interactions involving rearrangements of molecular conformations cannot be observed using the conventional biophysical tools such as nuclear magnetic resonance (NMR) spectroscopy, transmission electron microscopy (TEM), circular dichroism (CD), or any type of fluorescence spectroscopy, since the aforementioned technologies are not intended to apply force to biomolecules, including for the study of elastic properties [20].

Combined molecular dynamics simulations have recently been widely used to study the significance of the specific conformational rearrangements of enzymes during their functioning (for example, [21]). Despite the improvement in modeling methods, important limitations remain. The main limitation is that modeling cannot reach the time scales of most enzymatic reactions. Therefore, it is necessary to develop new experimental methods capable of investigating the dynamics of enzymes during catalysis, providing new information that complements the results obtained using existing experimental approaches and molecular modeling methods.

Contrariwise, the atomic force microscopy (AFM) allows the analysis of molecules by imitating conditions close to the cellular environment, as well as the study of objects in high nanometer-scale resolution [22]. The underlying principle of AFM enables the high-precision control of applied forces and the monitoring of objects at the molecular level. As for enzymes, they are a convenient system for observing the functional properties of single-protein molecules, since most of them undergo conformational changes during a catalytic reaction. Therefore, AFM enables monitoring the functioning of single-enzyme molecules in a liquid medium (i.e., under conditions maximally close to the native ones).

## 2. The AFM Principle

The underlying principle of AFM measurements is based on the interaction between the scanning element (a cantilever or probe) and the sample surface on which the analyzed bio-object is adsorbed. The necessary condition for using AFM is the adsorption of the analyzed enzyme onto the atomically smooth surface, which implies that the protein properties on the surface differ from those of a molecule in the solution. However, the immobilized enzyme system is used in many biotechnological, biosensor, and medical diagnostic systems [23]. From the perspective of conformational characteristics and retaining the functional properties of biomolecules, it might seem that the need to use the surface is a drawback of AFM. However, numerous studies reporting the results of using such systems to solve bioassay-related problems [24] confirm the stability of immobilized biomolecules and the preservation of their structural and functional properties [25,26].

As mentioned above, in AFM, the surface is scanned using an AFM probe; in most commercial AFM probes, the tip has the shape of a pointed pyramid. The size of the sensitive zone of the probe (the tip curvature radius) is several nanometers, which is comparable to the size of most globular proteins, including enzymes. According to TEM, NMR, and X-ray data, the protein size ranges from 5 to 10 nm [27], while the minimum radius of curvature of the probe tip is 1 nm, providing high-resolution AFM imaging of the surface with the adsorbed protein.

The tapping mode is traditionally used for biological objects. This is a measurement mode based on the detection of changes in the characteristics of probe vibrations depending on the surface topography, usually used for the visualization of fragile objects such as proteins [28]. This measurement mode is preferred because it minimizes the impact of the probe on analyzed objects, preserving their structural and functional properties. It is also a mode that provides new structural and mechanical information about the enzymes being studied. Thus, when using the harmonic oscillator model, the tapping mode based on nonlinear properties can enhance the processes that are barely detectable in the static (contact) mode [28].

The advances in AFM are the result of the optimization of sample preparation methods [29,30,31] and imaging [32], as well as the continued mastery of the methodology and hardware [33]. At its early stages, AFM analysis could be performed only in the air. More recently, a technique suitable for single-biomolecule analysis in physiologically relevant solutions has been developed, making AFM a sought-after technique in the research into enzyme systems.

The most recent key innovations include the optical positioning system and the bio-AFM liquid cell, AFM based on the quantitative detection of parameters of the force–distance curve (FD-AFM), and high-speed AFM (HS-AFM). Most of these modes are mutually complementary and are used in combinations [34,35]. The AFM methods can traditionally be divided into two large groups: AFM imaging and AFM-based force spectroscopy (AFM-FS). Table 1 summarizes the results of the study of enzyme systems using AFM. Our focus is on the results of studies for enzyme reactions, since there are not many such studies yet, in contrast to the AFM studies of the mechanistic properties of individual enzyme molecules.

## 3. AFM-Based Force Spectroscopy

AFM-FS techniques applied for mapping the interaction force under different loading conditions are often referred to as dynamic force spectroscopy [48], which is a separate research area for studying the elastic, adhesion, and denaturation properties of protein molecules.

**Figure 1:**
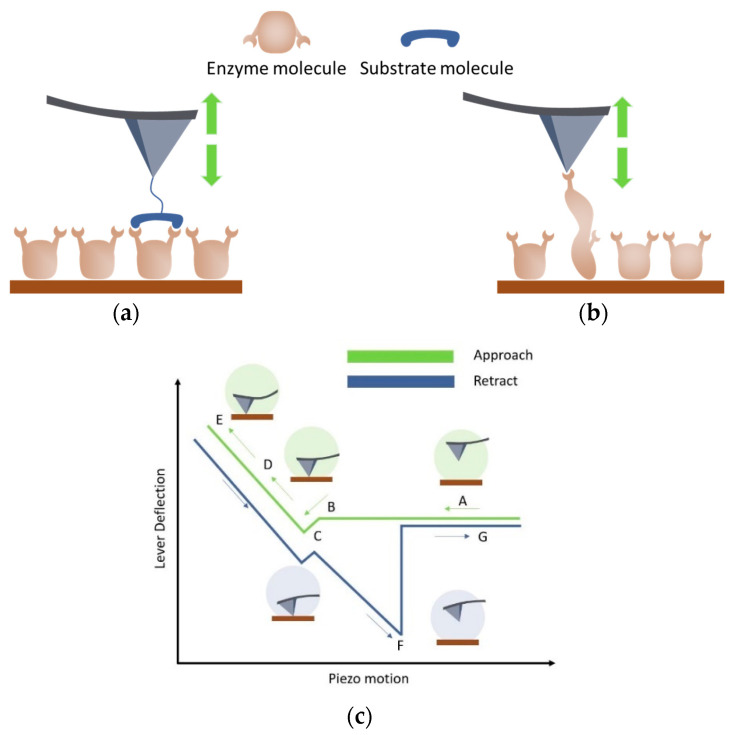
Scheme of data acquisition during measurements in the AFM-FS mode. The probe approaches and is retracted from the surface (green arrows). An enzyme is immobilized on the surface, a substrate molecule is attached to the probe tip (**a**), or the probe is not modified and the “force-clamp” technique is used (**b**). The dependence of the ‘lever deflection’ on the position of the piezoelectric element is recorded, which ensures the convergence of the probe and the surface. The “lever deflection” signal can be converted into a force value with high accuracy; the recording “piezo-motion” parameters allow us to determine the time of approach and the retraction of the probe from the object. The applied force, probe delay time, approach, and retraction speeds are recorded with high accuracy. A schematic representation of the force–distance curve is shown in (**c**). At large distances (A), the probe and the surface are far from each other, and, therefore, the probe deflection is not measured. As it approaches, the probe begins to feel long-range interactions, mainly of electrostatic and Van der Waals origin (B). The interaction of the surface and the probe tip may be reflected as a jump to the contact (C) in the case of attraction. A further approach leads to the deflection of the probe as it is physically in contact with the surface (D); further compression of the probe on the surface leads to further probe deflection. During the reverse movement of the probe, hysteresis (F) is observed, since the probe tip interacts with the surface (or enzyme molecule). Depending on the nature of the interaction and the presence of the different force levels of the interactions, the critical adhesion force (F) changes, and characteristic steps appear in the region (C–F). The careful processing of AFM data and a well-prepared experimental design allow obtaining information about intermolecular interactions (in case (**a**)) or about enzyme globule folding/unfolding and conformational rearrangements in the enzyme globule (**b**). Once the probe overcomes the adhesive force, it detaches from the surface (G).

Most studies focus on single-enzyme molecule systems using AFM-FS that deal with protein folding/unfolding. The principle of plotting force curves is based on monitoring probe deflection and piezoelectric element displacement during the cycle of the AFM probe approach and release with respect to the sample surface [49]. Graphically (Figure 1), the force curves show the applied force as a function of the distance between the probe tip and the sample. The investigation of the dynamic sub-angstrom-scale rearrangements of atoms involved in catalysis is an experimentally challenging problem [36].

Due to the feasibility of modifying the AFM probe surface and immobilizing molecules on it, AFM-FS can be used to investigate intermolecular interactions in the enzyme system (Figure 1a). In paper [50], the results of the studies of the interaction between lignin and an enzyme are considered. Lignin is a complex polymer that inhibits the enzymatic conversion of cellulose to glucose in lignocellulosic biomass for biofuel production. Cellulase enzymes irreversibly bind to lignin, deactivating the enzyme and reducing the overall activity of the hydrolysis reaction solution [38]. One of the participants in the enzymatic reaction must be attached to the tip of the AFM probe, while the other biomolecules are immobilized on the substrate. The paper [50] summarizes studies using probe-attached receptor molecules to study enzyme–lignin interactions at the single-molecule level.

Conformational rearrangements in the enzyme globule occur during the catalytic cycle due to covalent bond formation/cleavage and atomic rearrangement [51] (Figure 1b). The stiffness of a covalent bond is ∼10 nN/Å; the distance in the transition state for a chemical reaction is typically a fraction of an angstrom, so a respective impact of the probe (in the range of ~100 pN to ~1 nN) on the bond is required, which is applicable in the AFM modes [36]. In the modes utilizing a “force clamp” immobilized on the object surface, the force applied to the protein globule can be varied with an accuracy of several hundred piconewtons [52]. The deflection of the probe and the applied force are kept constant by a highly sensitive electronic feedback system [53]. In this approach, forces are applied directly to the disulfide bond in the substrate.

**Figure 2:**
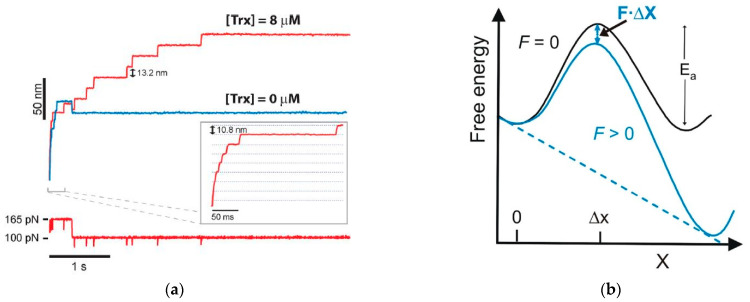
(**a**) At an applied force of 165 pN, a series of 10.8 nm steps is detected, reflecting the rapid unfolding of the modules to the disulfide bond. If a reducing agent such as the Trx enzyme is present in the solution (red curve), a second series of 13.2 nm steps is detected. These correspond to the reduction in disulfide bonds and the subsequent release of residues 32 through 75. In the absence of a reducing agent in solution, no reduction occurs (blue curve) [36]. (**b**) Diagram of the energy landscape for the thiol/disulfide exchange reaction under force, where ∆x is the distance to the transition state of the reaction. Figures adapted from [36].

Alegre-Cebollada et al. [36] demonstrated the feasibility of quantifying the effect of the applied force on the enzymatic cleavage of covalent bonds (Figure 2). The results of the application of AFM-FS in the study of the mechanism of disulfide bond reduction by various enzymes of the thioredoxin (Trxs) family are considered. Thioredoxins are reductases that catalyze cysteine–thiol–disulfide exchange reactions. There is evidence that the thioredoxin system is involved in the processes of aging, carcinogenesis, the regulation of proliferation, and apoptosis [54].

The article [36] considers in detail the specific requirements necessary for the application of AFM to an enzyme system. The dependence of the reduction strength of disulfide bonds has been studied for both various chemicals as well as for different Trxs. The work [36] has shown that the enzymatic reaction depends on probe force, which is related to sub-angstrom scale rearrangements in the thioredoxin enzyme and substrate during catalysis [55,56]. To study the process of disulfide bond reduction, two measurement schemes are used in which the applied force is varied. Applying a force of 160–190 pN for 0.3–1.0 s unfolds the domains of the polyprotein. Forces greater than 1 nN are required to break covalent bonds. Therefore, individual unfolding events can be clearly detected as a 10.8 nm step increase in the length of the polyprotein, resulting in well-defined steps in the length-time plot. The shape of the dependences serves as a well-defined fingerprint that definitively distinguishes the polyprotein of interest from any other spurious interactions. Successive unfolding events under the action of force allow the detection of disulfide bonds that have been closed in the protein. The authors showed that the dependence of the reaction rate on the force provides new knowledge into the dynamics of the enzyme and substrate during catalysis. Using the parameter ∆x (i.e., the distance to the transition state of the reaction), which can be determined from the obtained force–distance curves, it is possible to assume the presence of the spatial rearrangements of the polypeptide chain regions in the transition state of the reaction. The authors obtained information about the geometry of the transition state by using chemical reagents to cleave the disulfide bond. The authors suggest that the information obtained about the transition state is independent of the lifetime of the enzyme in this state. The advantage is that no matter how short- or long-lived the transition state is, it can always be detected by varying the strength of the impact. The authors emphasize that these rearrangements can be determined on a sub-angstrom scale only using AFM and are unattainable by other modern experimental techniques.

AFM has great potential to study the interaction between lignin and an enzyme under conditions close to physiological ones. Due to the high molecular weight of cellulase, steric hindrances for intermolecular interactions occur when modified on the AFM probe. Most cellulases consist of two domains: the catalytic domain (CD) and the cellulose-binding module (CBM), connected by a highly glycosylated flexible linker. CBM plays an important role in the binding of lignin to cellulase, revealed by molecular dynamics simulations [57]. Since cellulase is not suitable for plate modification, CBM is commonly used to represent cellulase enzymes and to functionalize AFM plates to study the lignin/cellulose-enzyme system at the nanoscale. The authors of this work [50] point out that when studying the force interaction for an enzyme to be immobilized on a probe, the choice of crosslinker is critical. The modification of biomolecules should result in the fact that during the studies, only one enzyme at the tip can contact another molecule modified on the surface [35]. Among the methods, using AFM-FS, which is capable of detecting the bond strength of individual molecules, is often used. Arslan et al. conducted a series of studies [37] on the nanoscale interaction between lignin and CBM (used as a representative enzyme model) (Table 1). It was found that electrostatic and dipole–dipole forces mainly create the interaction between CBM and lignosulfonates. Hydrophobic forces and Lifshitz–van der Waals forces are characteristic of the interaction of kraft lignin and CBM. Organosolv lignin showed the weakest non-productive bond with CBM. Qin et al. [38] studied the interaction between lignin and cellulase based on the adhesion of a substrate-immobilized enzyme to a substrate molecule immobilized on the probe. The immobilization must maintain sufficient mobility and freedom of orientation to facilitate recognition as well as properly bind enzymes to the surface through covalent or noncovalent bonds. The results of this study showed that the measured adhesive forces between lignin and cellulase were, on average, 45% higher than those between hydroxypropyl cellulose and cellulase, demonstrating the specific nature of the binding in the enzyme–substrate complex.

Unfortunately, AFM-FS usually requires longer data acquisition time compared to AFM imaging, thus limiting the wide use of this approach. Moreover, this mode is incapable of detailing a region of the enzyme globule for which the parameter is recorded. As a result, AFM-FS can provide additional information about the mechanism of an enzymatic reaction and changes in the structural properties of a molecule during the reaction at the single-molecule level, but the kinetic parameters of the reaction cannot be determined using this technique.

## 4. AFM Imaging

The investigation of enzyme systems based on the AFM imaging data (i.e., surface topography measurements) was started quite a long time ago [39]. However, although AFM has been successfully used to visualize protein structures, early studies focusing on the dynamics of changes in protein globules during a reaction were hampered by the low scanning speed of AFM.

The scan rate of standard equipment was, on average, one line per second, so it took up to several minutes to acquire a single image of the desired resolution, whereas biological reactions proceed at the millisecond and shorter time scales. Dynamic processes in protein molecules occur at several time scales: from tens of femtoseconds to hundreds of seconds [58]. Fluctuations or any other conformational motions in proteins are related to time scale (e.g., vibrations occur within hundreds of femtoseconds, while large motions such as domain motions in proteins occur at the millisecond time scale [58]).

Scanning speed is a significant factor affecting the research results and defining the range of problems that can be solved. AFM scanning is accompanied by both sample deformation and image displacement caused by the system drift, especially when scanning in solutions. It is important to take into account data asynchrony, as the data for all the pixels in the image are acquired not simultaneously but with a sequential time delay upon the linewise movement of the probe (for probe scanning) or the specimen stage (for specimen scanning). Accordingly, one can imagine that the slow scanning of a rapidly changing object will result in low spatial resolution [59]. Biological molecules undergo translational diffusion, rotational diffusion, and conformational changes related to their functions, which affect the morphology of the observed objects and, therefore, the apparent spatial resolution of the AFM image. Therefore, an improvement in the temporal resolution of AFM is key to imaging dynamic and mobile biological molecules and clearly resolving their detailed characteristics [60].

**Figure 3:**
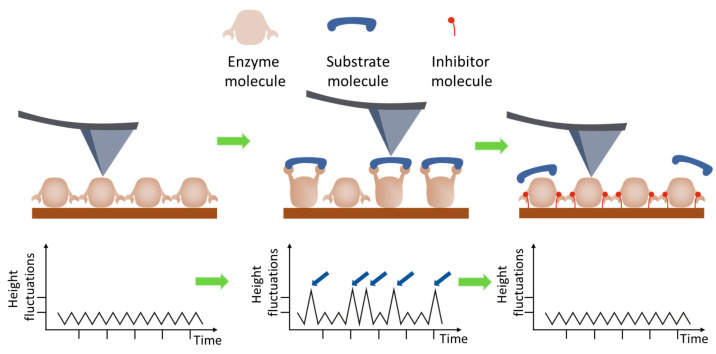
The scheme of measurements in AFM imaging mode to observe protein height oscillations during the catalytic activity of proteins, as proposed in [39]. The probe is installed above a selected surface area containing a layer of enzyme molecules. In the first stage, the height of the objects is recorded under the conditions of an enzyme reaction without the necessary component—the substrate (**left panel**); the initial level of oscillation of the height of the molecule is measured. In the second stage, the substrate is added to the liquid medium, the enzyme reaction is initiated, and the level of oscillation increases (**middle panel**). For control, an inhibitor is added to the system, and the enzyme reaction does not proceed; the level of the height oscillation remains at the background level (**right panel**). These measurements indicate that the observed jumps (**middle panel**, plot image, small blue arrows) in the measured height in the presence of substrate are probably due to the enzymatic activity of lysozyme.

In the work [39], the height fluctuations of lysozyme molecules were studied using AFM in the tapping mode in liquid (Figure 3).

**Figure 4:**
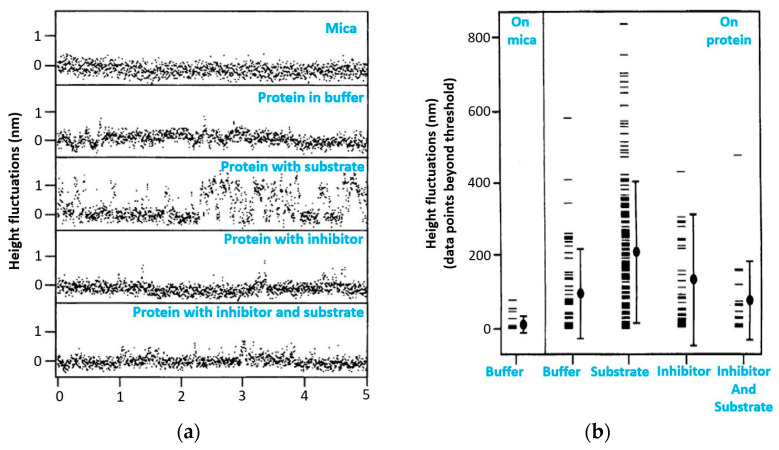
(**a**) Monolayer height variations in lysozyme molecules adsorbed on mica measured by AFM. The data were recorded on mica or on lysozyme in buffer, on lysozyme in buffer containing the substrate 4-methylumbelliferyl-N,N′,N″-triacetyl-chitotriose (~10 µM), in buffer containing the inhibiting substance N,N′-chitobiose (~20 µM), and in buffer containing both substances. Spikelike jumps appear in the height signal. The apparent height of these jumps is on the order of 1 nm. (**b**) Comparison of a dataset of 271 points from six different preparations. The graph shows the points that exceeded the mean value of the data by more than 0.5 nm. The mean and standard deviation are indicated by the filled circle and vertical line. Figure adapted from [39].

The essence of this work [39] is that the height measurements were made by fixing the probe on a lysozyme monolayer without scanning for 32 s. This method made it possible to detect the vibrations of lysozyme molecules in the presence of a substrate in the system at 1 nm, but vibrations were absent in other systems, such as a protein buffer solution, a protein with an inhibitor, and a protein with an inhibitor and a substrate (Figure 4). The authors of the article associate vibrations with possible conformational changes in lysozyme caused by hydrophobic interactions during hydrolysis. It was calculated that a force of 50 pN is required to bend the cantilever by 1 nm, and the energy associated with this bending is approximately 0.1 eV. The enthalpy of hydrolysis is about 0.5 eV per substrate molecule, which is sufficient to bend the cantilever. These measurements indicate that the observed jumps in the measured height in the presence of substrate are probably due to the enzymatic activity of lysozyme.

**Figure 5:**
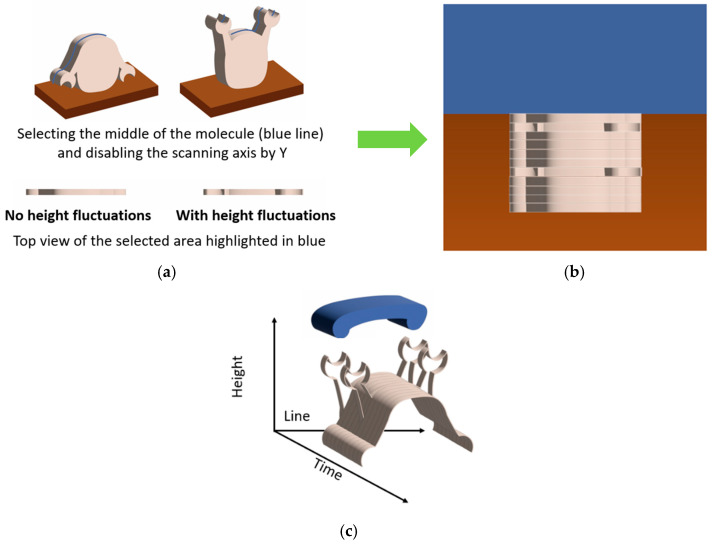
AFM data for the registration of molecular height oscillations during functional activity; an approach was applied in [40]. The height oscillations of individual molecules can be recorded at each stage of the catalytic cycle of the enzyme. (**a**) The first step is the registration of surface topography. The area of interest for the visualized object is selected; (**b**) the second step is topography registration along Ox, and the slow Oy axis was turned off; (**c**) 3D visualization of the second-step results. The Oy axis corresponds to a time of observation for each molecule. The trajectory of the fluctuations of the maximum height of this molecule as a function of time, h_max_(t), may be determined in the image of the sections of a single molecule.

In paper [40], cytochrome P450 activity was studied using the measurement principle similar to that reported by Radmacher et al. An approach (Figure 5) has been developed to measure the activity of the single oligomers of the heme-carrying enzyme, cytochrome P450 CYP102A1, based on the AFM imaging data. The amplitude of the height oscillations of single-molecule enzymes involved in the catalytic cycle was shown to be twice as high as the height oscillation amplitude of the same enzymes in the inactive state. It was also demonstrated that the amplitude of the height oscillations of the CYP102A1 protein globule is temperature dependent, and the peak in this curve was observed at 22 °C. The activity of a single CYP102A1 molecule expressed as a unit amplitude of the height oscillations of the protein globule per unit of time was 5 ± 2 Å/s. This process was recorded based on changes in heights (Oz axis, labeled height in schematic Figure 5c) over the observation time for each molecule (Oy axis, labeled time in schematic Figure 5c).

The kinetics of the enzymatic reaction were also studied by Balashev et al. using the AFM measurements of the topography [41]. This study was based on the evaluation of the occurrence and the growth rate of the defects of the dipalmitoyl phosphatidylcholine (DPPC) degradation layers induced by the lipase enzyme. It was demonstrated that an analysis of sequential AFM images allows one to assess the degradation rate of the adsorbed bilayer, which was attributed to the formation of a product of enzymatic hydrolysis. In this study, the authors relied on an assessment of the changes in the heights of the substrate layers over time, which is not quite suitable for studying the enzyme systems conventionally using low-molecular-weight substances, which are difficult to detect by AFM as substrates.

Also, in our review, it is worth mentioning that one of the articles cited in [50] is devoted to the structural changes in the lignocellulosic substrate in situ during the enzymatic reaction. Lambert et al. proposed using in situ AFM to visually determine the enzymatic hydrolysis of lignocellulosic films with different lignin contents [42]. This paper proposes a strategy to gain insight into the resistance of lignin to enzymatic hydrolysis. Cellulose nanofibril (CNF) lignocellulose films with increased lignin content (up to 40%) were prepared. In situ measurements were performed in real time using atomic force microscopy (AFM) during hydrolysis, and the results were compared with biochemical analyses. Based on the results of the work, the authors emphasized the importance of lignin content and the mutual orientation of CNF and lignin for the efficiency of hydrolysis. An original quantitative analysis of in situ measurements with time-lapse measurements is proposed to visualize the in situ deconstruction of complex lignocellulosic substrates.

A significant breakthrough in the study of biological processes was made as a set of tools has been achieved with the development of a number of tools and the design of high-speed AFM-based (HS-AFM) systems. Several improvements aimed at rapid scanning while focusing on the investigation of biological specimens have been implemented [61,62,63]. It should be noted that when discussing the speed of HS-AFM imaging, several possible definitions of speed have to be distinguished, such as (a) the image acquisition time (frames/s) and (b) the scanning speed of a probe (m/s). Since we further discuss the use of HS-AFM to study the dynamics of changes in protein globule morphology and their relationship to catalytic activity, the lag time between image acquisitions is more significant than the scanning speed of a probe [64].

The use of small probes with resonant frequencies lying in the megahertz range was one of the key technical solutions [65]. The design of rigid and compact piezoelectric scanners in combination with the development of control techniques has significantly improved the AFM imaging technology [66]. The HS-AFM equipment currently allows scanning at a frame rate of ~33 frames/s and temporal and spatial resolutions comparable to the dynamic analysis of biospecimens [67,68]. The details of the setup and the improvement of its components have recently been thoroughly reviewed in detail in [69,70].

HS-AFM imaging is an approach that has enabled the real-time visualization of biological macromolecules during their functioning. The temporal resolution is typically less than 100 ms; the lateral and vertical spatial resolutions are 2–3 nm and ∼0.1 nm, respectively [60]. However, the level of detail obtained in HS-AFM experiments is critically dependent on the spatial and temporal resolutions of the system. HS-AFM has been used to directly observe reactions, such as the formation of the enzyme–substrate complex and protein folding by chaperones, to study caseinolytic peptidase B protein homolog (ClpB) [43], ATPase histone chaperone Abo1 [44], and V1-ATPase [45]. HS-AFM made it possible to detect conformational changes in a molecule upon visualization in solutions, but it is worth mentioning that all the proteins were well immobilized on the mica surface. The choice of an immobilization method is of crucial importance for imaging molecules while preserving their actual structural and functional features and not preventing their conformational changes.

An example of employing HS-AFM is the study of the structural dynamics of laminin-111 and laminin-332 under physiological conditions [46]. The results demonstrate that the coiled-coil domain of laminin-332 is highly dynamically bent around a defined central molecular hinge, whereas the coiled-coil domains of laminin-111 retain their relatively stable S-shaped configuration. Furthermore, structural fluctuations in the cluster of C-terminal LG domains of laminin-111 and laminin-332 between the compact and open conformations were detected, which may play a role in the regulation of the binding of adhesion receptors. Thus, it was demonstrated that HS-AFM can reveal isoform-specific conformational changes occurring in different laminin domains, thus providing new insights into the dynamic structure and specific assembly of laminin affecting the functional properties of the chain.

**Figure 6:**
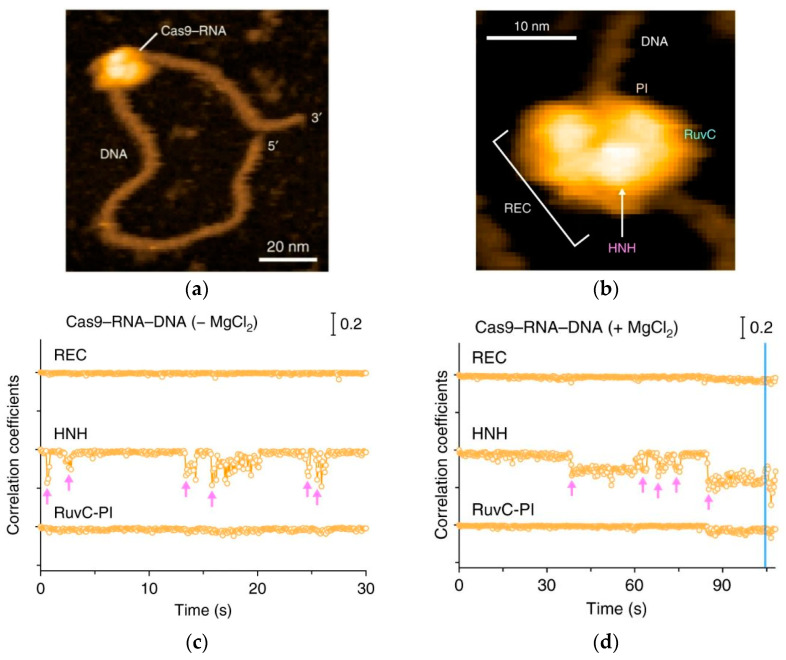
(**a**) Complex assembly of Cas9–RNA–DNA; the scale bar is 20 nm. (**b**) Close-up view of a representative HS-AFM image of Cas9–RNA–DNA. The scale bar is 10 nm. (**c**) Time courses of correlation coefficients for the individual domains between the sequential HS-AFM images of Cas9–RNA–DNA in the absence of MgCl_2_. The HNH domain fluctuations are indicated by magenta arrows. (**d**) The time courses of the correlation coefficients for the individual domains between the sequential HS-AFM images of Cas9–RNA–DNA in the presence of MgCl_2_. The HNH domain fluctuations are indicated by magenta arrows. The release of the cleavage product is indicated by a blue line. Figure adapted from [47].

Another example of using HS-AFM is the visualization of target DNA cleavage by the CRISPR/Cas9 complex during the life cycle [47]. The AFM data have provided data about the functions of the CRISPR/Cas9 complex, including complex assembly, target DNA search, chain cleavage, and product release, which have improved the understanding of the mechanism of action of the gene-editing tool. Of particular interest is that this study has demonstrated the oscillation of the Cas9 nuclease HNH domain, which is responsible for site cleavage, upon binding to the target DNA, and showed differentiation between active and stable closed conformations of a single Cas9 molecule and the Cas9–RNA complex on the mica surface (Figure 6).

**Figure 7:**
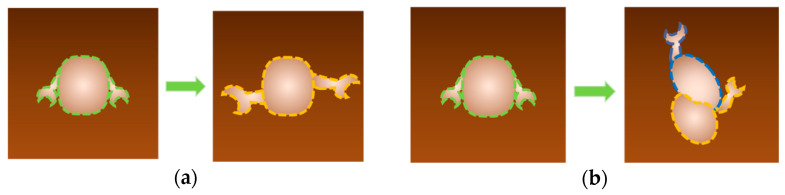
Scheme of interpretation of data obtained using high-speed AFM imaging (HS-AFM). During the catalytic cycle, a series of images is recorded, the target object—an enzyme molecule or some domain—is isolated, and the morphology of the object is analyzed. There are several methods for analyzing the morphology and motion of a protein globule using AFM data. General conformational changes for the observed molecules can be determined from their size (e.g., by calculating their circumference, a parameter indicating how circular the contour of the observed molecule is) (**a**) or by observing the trajectories of the target structures (**b**).

Special attention should be paid to the analysis of the data obtained by high-speed AFM imaging. There exist several methods for analyzing the morphology and motion of a protein globule according to the AFM data (Figure 7). The overall conformational changes for the observed molecules can be determined from their size (e.g., by calculating their circumference), a parameter indicating how circular the contour of the observed molecule is, which is defined as Equation 4πS/L2, where L and S are the contour length and the area enclosed by the contour, respectively [43,71]. When molecule parts change their shape and/or mutual arrangement, conformational changes can be detected by tracking the motion trajectory of a certain section of the moving domain(s)/cluster(s) [68,72]. The histograms of the distances traveled by certain domains allow one to estimate how significant the conformational changes that a molecule undergoes during the observation period are [72]. In all cases, mathematical and statistical analyses based on the observations of a large number of molecules (usually hundreds of molecules) are required to verify the objective conclusion. For example, it has been demonstrated that HS-AFM imaging of single ERdj5 molecules revealed multiple cluster orientations and the highly mobile nature of the C-terminal cluster compared to the N-terminal cluster [72]. The authors analyzed the movement of ERdj5 clusters by tracking trajectories and plotting a histogram of their travel distances. To assess the conformational diversity of ERdj5 molecules, Okumura et al. [73] calculated the circularity for each observed molecule and plotted a histogram. They demonstrated that the average circularity of the wide-type ERdj5 wild type (WT) (0.67) was lower than that for the mutant proteoforms in which the cluster orientation was fixed as form I (0.76) or form II (0.71), and the half-width of the Gaussian fitting curve for WT was the largest among these three variants. This indicated highly dynamic conformational changes for WT and the most circular shape for the mutant proteoform I, as evidenced by the cyclicity revealed by tracking the cluster trajectory [72].

The analysis of the AFM imaging data acquired by HS-AFM differs significantly from the analysis of the previously used data obtained using the standard equipment. For example, in the study mentioned previously [40], the oscillations of the height of individual CYP102A1 molecules were recorded at each stage of the catalytic cycle of the enzyme during lauric acid hydroxylation using the adapted method described in ref. [39]. The principle of AFM data acquisition is shown in Figure 4. The AFM images of at least ten different molecules of the enzyme were recorded at each stage. First, the surface topography (Figure 5a) was recorded by selecting an object in the image whose height corresponded to that of a protein globule. Then, during AFM visualization, scanning along the slow Oy axis was turned off (Figure 5b). Hence, an image was obtained as “sections” for each visualized molecule (Figure 5c); i.e., a time sweep of one line of a topography image section was recorded. The trajectory of fluctuations of the maximum height of this molecule as a function of time, h_max_(t), was determined in the image of the sections of a single molecule. For a comparative analysis of the trajectories of different molecules, their linear fitting was performed. The value (the amplitude of height fluctuations for a single molecule over a time interval) was calculated using the result of such an action. Unfortunately, the observation time for each molecule did not exceed 30–40 s. It was not possible to record the signal from a single molecule for a longer period of time, presumably due to substrate drift. Therefore, it was impossible to detect changes in molecule height for a single molecule throughout all the stages of the catalytic cycle, so the data averaged with respect to the results of measuring different molecules at different stages of the catalytic cycle are presented in this study.

**Figure 8:**
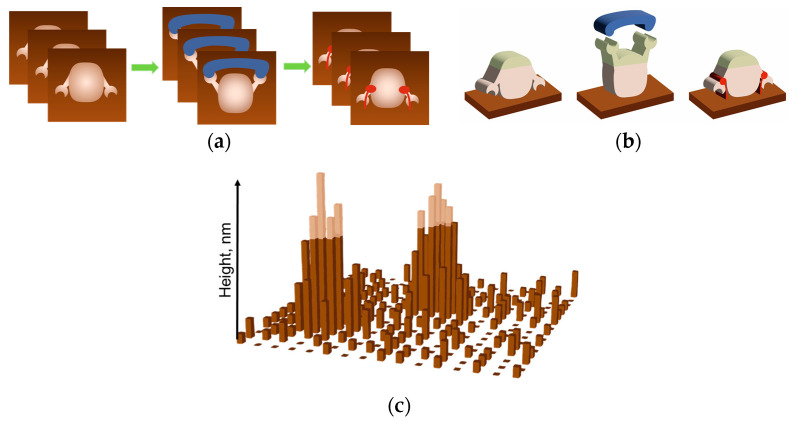
AFM data for recording molecular height variations during functional activity using the NanoTrack^TM^ positioning feature. (**a**) The first step is to register a collection of images for individually selected objects. A collection is created for each catalytic cycle study. (**b**) The second step is to select a region of interest for the visualized object (marked with a green outline). (**c**) The third step is data processing. The height of each pixel and the relationship between them can be determined using a dedicated script [74]. Only the tip of the molecule is considered, and the height of pixels above a certain level (light area in (**c**)) is used for the calculation. It is assumed that the ratio of the height of the pixels in the image of a single molecule can be related to the activity of the molecule during operation. A comparison of data obtained for different catalytic studies allows us to obtain relationships between height and activity at the single-molecule level.

In the work [40], measurements were conducted using a Dimension 3100 atomic force microscope (Bruker, Santa Barbara, CA, USA). Today, there is a novel modification of the equipment, the Dimension FastScan atomic force microscope (Bruker, USA), having a unique NanoTrack^TM^ positioning function. The scan area can be positioned and retained for individually selected objects within a single AFM image (Figure 8a). A collection of images is acquired; their analysis for each scan allows one to determine the parameters of a visualized enzyme globule during its functioning. Thus, it is possible to highlight the tip of the molecule (Figure 8b) or an area of interest for the visualized object, which is presumably related to the active site of an enzyme molecule. It is possible to track the maximum height of a protein globule over a long period of time [74] by registering the height of each pixel and determining the ratio between them.

## 5. Combining AFM with Infrared Spectroscopy

The enhancement of AFM with additional techniques such as infrared spectroscopy (IR) is effectively used to characterize the structural properties of biologically significant materials at the nanoscale. For example, the combination of IR with AFM (IR-AFM) has been successfully used to study amyloid aggregation processes [75], the local broadband spectra of ferritin complexes and insulin aggregates, which can be interpreted in terms of their α-helical and/or β-sheet structure [76].

IR-AFM methods such as scanning Near-Field Scattering Optical Microscopy (s-SNOM) and Peak Force Infrared Microscopy (PFIR) take full advantage of the reduced tip–sample interaction region, achieving a spatial resolution of 10–20 nm without the use of specialized labels. The advantage of these methods is that the spatial resolution is not limited by the diffraction limit (as in classical IR) but by the geometry of the AFM tip and its radius of curvature. In addition to high spatial resolution, the strong near-field enhancement of the tip also provides chemical sensitivity. However, when analyzing complex biological samples containing complexes and groups of biomolecules, the IR spectrum may be a mixture of all the signals obtained [77]. This factor complicates the registration of a signal at the level of single molecules. The isolation of the target spectrum from the general spectrum may require the use of IR labels, which can affect the functional activity of biological objects, in particular, during the catalytic cycle of enzymes. Another disadvantage of IR-AFM methods is that most studies in biological systems have been carried out in the air using dried samples, which limits the space for studying enzymes during the catalytic cycle. It is due to the fact that the implementation of IR-AFM in an aqueous environment is limited to strong signal attenuation in liquid in the mid-IR range [78].

However, the technologies for using IR-AFM in liquids (fundamental, biological, and medicinal) are actively developing and are quite promising. For example, in [79], the authors demonstrated the potential for nanoimaging of s-SNOM in the liquid and conformational identification of catalase nanocrystals, as well as the spatial and spectral analysis of biomimetic peptoid sheets with monolayer sensitivity and chemical specificity at the few-zeptomoles level by additional plasmon field enhancement using metal nanoplates. Also, the successful implementation of the IR-AFM method in liquid is described in [80], where nano-FTIR spectroscopic imaging in liquid was experimentally demonstrated.

In addition, the development of IR-AFM methods can be facilitated by combining mid-IR spectroscopy with high-speed AFM. Since HS-AFM usually uses a shortened cantilever with high resonant frequencies (for example, in MHz), this can potentially contribute to improving the resolution. It is expected that the chemical visualization of the rapid changes in enzyme function in a physiological environment can be successfully implemented and will help to provide additional data for the study of enzyme systems.

It is possible that the development of new technologies in the field of IR-AFM in liquids, will lead to more work being devoted directly to the study of enzymes during the catalytic cycle at the level of individual molecules.

## 6. Conclusions

The results obtained by various research groups confirm the possibility of using AFM for the visualization, functional, and mechanistic properties of enzymes. Depending on the task, different measurement modes can be used. The need to immobilize the target objects on the surface limits but does not fully restrain the scope of this technique. The results of many studies show that the functional properties of molecules on the surface are preserved. Moreover, the immobilized enzyme system in some cases can become an object of study, since it is used in biosensors or biotechnological workflows. To date, there are not many examples of studying enzyme systems, i.e., the parameters of kinetic processes and not the properties of individual enzyme molecules. Particular attention is paid to the use of HS-AFM, but this equipment, unfortunately, is quite unique and complex. However, this review has shown that standard equipment can be successfully used to study enzyme systems. Understanding the mechanism at a new level of individual molecules is also very important for the development of the industry of producing synthesized enzymes, which are intended to reduce the cost of biotechnological processes. But, as a rule, synthesized enzymes have lower activity than natural analogs. Perhaps the answer to this question can be obtained using AFM as well.

## Figures and Tables

**Table 1:** Summary of the results of the studies of enzyme systems using AFM.

AFM Mode	Enzyme System	Parameter	Reference
AFM-FS	Thioredoxin family	Mechanism of reduction in disulfide bonds	Alegre-Cebollada et al. [36]
	Cellulase (CBM 1, CBH I, and Trichoderma reesei)	The bond strength of individual molecules	Arslan et al. [37]
	Cellulase	Comparison of the adhesion forces between cellulase and lignin with those between cellulase and cellulose and the examination of the moiety groups involved in cellulase binding to lignin	Qin et al. [38]
AFM imaging	Lysozyme	Height fluctuations of lysozyme protein molecules	Radmacher et al. [39]
	P450 CYP102A1	Height fluctuations of protein molecules	Ivanov et al. [40]
	Lipase	Layers degradation induced by the lipase enzyme	Balashev et al. [41]
	Lignin	Layers degradation of lignocellulose films during hydrolysis	Lambert et al. [42]
AFM imaging (HS-AFM)	Caseinolytic peptidase B protein homolog (ClpB)	The dynamics of changes in protein globule morphology and their relationship to catalytic activity	Uchihashi et al. [43]
	ATPase histone chaperone Abo1		Cho et al. [44]
	V1-ATPase		Maruyama et al. [45]
	Laminin-111 and laminin-332		Akter et al. [46]
	Cas9 nuclease		Shibata et al. [47]

## Data Availability

Not applicable.
